# Phylogeography of the highly invasive sugar beet nematode, *Heterodera schachtii* (Schmidt, 1871), based on microsatellites

**DOI:** 10.1111/eva.12719

**Published:** 2018-10-24

**Authors:** Jiyeon Kim, Gang Ni, Taeho Kim, Jae‐Yong Chun, Elizabeth M. A. Kern, Joong‐Ki Park

**Affiliations:** ^1^ Division of EcoScience Ewha Womans University Seoul Korea; ^2^ Freshwater Biodiversity Research Division Nakdonggang National Institute of Biological Resources Sangju Korea; ^3^ Animal and Plant Quarantine Agency Gimcheon‐si Korea

**Keywords:** approximate Bayesian computation, cryptic lineage, cyst nematode, ghost population, invasion genetics

## Abstract

Plant‐parasitic nematodes (PPNs) threaten crop production worldwide. Yet few studies have examined their intraspecific genetic diversity or patterns of invasion, critical data for managing the spread of these cryptic pests. The sugar beet nematode *Heterodera schachtii*, a global invader that parasitizes over 200 plant species, represents a model for addressing important questions about the invasion genetics of PPNs. Here, a phylogeographic study using 15 microsatellite markers was conducted on 231 *H. schachtii* individuals sampled from four continents, and invasion history was reconstructed through an approximate Bayesian computation approach, with emphasis on the origin of newly discovered populations in Korea. Multiple analyses confirmed the existence of cryptic lineages within this species, with the Korean populations comprising one group (group 1) and the populations from Europe, Australia, North America, and western Asia comprising another (group 2). No multilocus genotypes were shared between the two groups, and large genetic distance was inferred between them. Population subdivision was also revealed among the populations of group 2 in both population comparison and STRUCTURE analyses, mostly due to different divergent times between invasive and source populations. The Korean populations showed substantial genetic homogeneity and likely originated from a single invasion event. However, none of the other studied populations were implicated as the source. Further studies with additional populations are needed to better describe the distribution of the potential source population for the East Asian lineage.

## 1 INTRODUCTION

Nematodes represent one of the most numerous and widespread groups of animals in the world. About 10% of the 20,000 described nematode species are phytohelminths (Malakhov, 1994), plant‐parasitic nematodes (PPNs) that reduce crop yields and cause global agricultural losses of $157 billion annually (Abad et al., 2008). The sugar beet PPN *Heterodera schachtii* (Schmidt, 1871) is one of the world's major pests of agricultural crops. It parasitizes more than 200 plant species within 95 genera from 23 plant families including Amaranthaceae (many species of *Beta* and *Chenopodium*), Brassicaceae, and Solanaceae (Steele, 1965).

**Figure 1:**
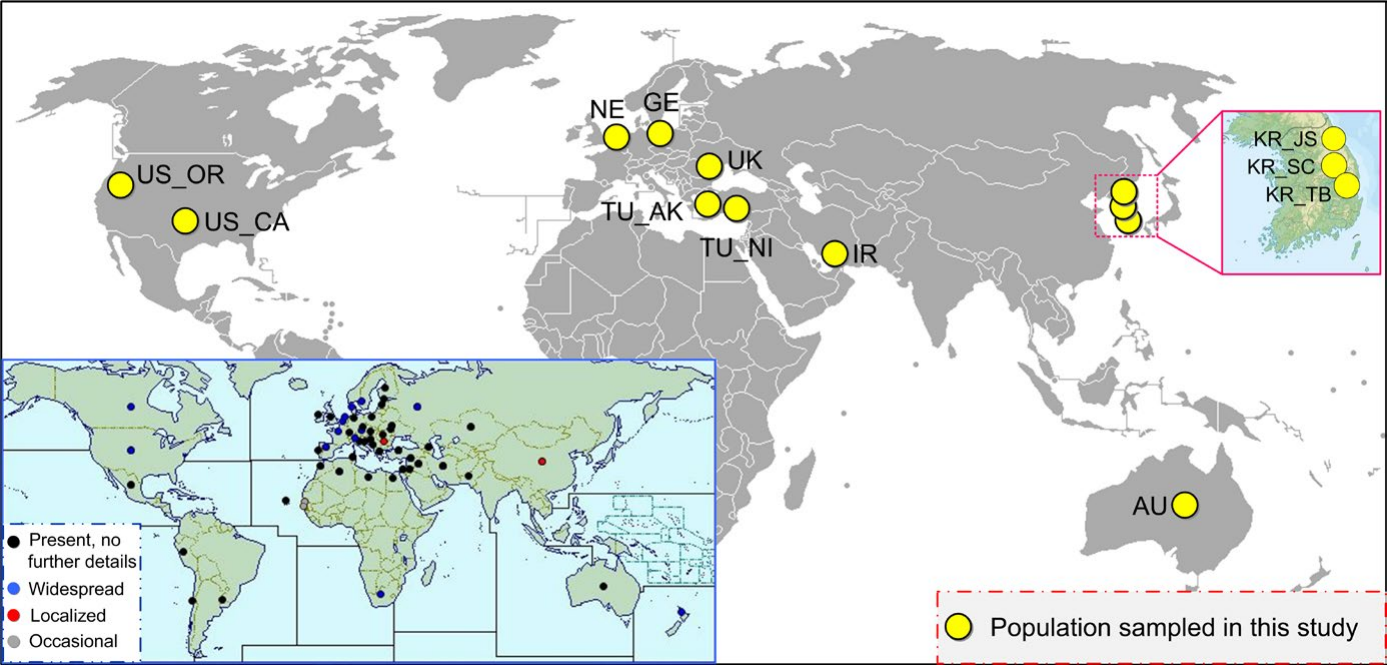
Global map showing the sampling locations of *Heterodera schachtii*. Populations are labeled with abbreviations as given in Table 1. The insert map indicates the global distribution of *H. schachtii* according to the records at www.cabi.org (accessed on June 2017)

*H. schachtii* was first recognized as a plant pathogen in 1859 when it caused stunted growth and declining sugar beet yields in Germany (Schacht, 1859) and was later described in other beet‐growing areas of Europe (e.g., Petherbridge & Jones, 1944; Greco, Brandonisio, & De Marinis, 1982; Steele, Toxopeus, & Heijbroek, 1983). This invasion‐prone species can easily spread undetected, in part due to its persistent dormant stage (microscopic cysts filled with eggs) that enables it to withstand desiccation for long periods (Subbotin, Mundo‐Ocampo, & Baldwin, 2010). At present, this nematode has been described from more than 50 countries and regions in six continents, covering all major agricultural production areas of the world (Subbotin et al., 2010) (Figure 1).

In 2011, *H. schachtii* infestations were reported in highland vegetable cultivation areas in Gangwon province, South Korea, on the roots of Chinese cabbage (*Brassica rapa*)—the primary ingredient of the traditional Korean food, kimchi (Kim et al., 2016). The pest then quickly spread to other healthy Chinese cabbage fields, causing increasing economic losses to the Korean Peninsula (Kwon, Shin, Kabir, Lee, & Lee, 2016). This relatively new invasion is particularly concerning because East Asia is the world's most populous area (Halpern et al., 2008), and high levels of travel and trade among countries increase the likelihood that *H. schachtii* will spread to the entire region.

Considering the significance that *H. schachtii* has for the economy and ecology, there is an urgent need to develop more effective management of this pest (Subbotin et al., 2010). Understanding its genetic diversity and structure and deciphering its invasion routes are central to monitoring and controlling its spread. Indeed, numerous studies have been conducted over the last few decades to evaluate the genetic diversity of *H. schachtii* populations, using diverse markers and approaches including PCR‐RAPD (Ambrogioni & Irdani, 2001; Amin, Subbotin, & Moens, 2003), AFLP (Madani et al., 2007), the ITS‐rRNA gene (Maafi, Subbotin, & Moens, 2003; Madani, Vovlas, Castillo, Subbotin, & Moens, 2004), and microsatellites (Plantard & Porte, 2004). However, previous studies had limited spatial coverage, used low‐resolution genetic markers, or employed inadequate statistical methods as discussed by Estoup and Guillemaud (2010). Genetic patterns of *H. schachtii* have never been fully described on a broad scale, and the discovery of new populations in Korea underscores the need for a better understanding of this species’ movement and population connectivity.

In this study, we address fundamental questions on the genetics and past movements of *H. schachtii* using an integrative phylogeographic approach. Phylogeography has for decades been a frequently applied tool for investigating evolutionary scenarios in invasive species (Bock et al., 2015; Karn & Jasieniuk, 2017). It has been successfully used to indicate potential source populations, reconstruct invasion routes, and test multiple introduction events in diverse species including blue mussels (Mathiesen et al., 2017), fire ants (Ascunce et al., 2011), and parasites (Criscione, Poulin, & Blouin, 2005). These studies have provided essential knowledge about the evolutionary dynamics of invasive species otherwise difficult or impossible to obtain by other means (Gotzek, Axen, Suarez, Helms Cahan, & Shoemaker, 2015).

Here, we analyzed specimens from four different continents including the recently discovered Korean populations, covering both assumed source and invasive populations, and then genotyped each of the 231 individuals using 15 newly developed microsatellite markers (Kim et al., 2016). Compared with other markers (e.g., ITS‐rRNA and COI) or fingerprinting strategies (e.g., PCR‐RAPD), microsatellites generally have high mutation rates that result in high levels of polymorphism (Pedreschi, Kelly‐Quinn, Caffrey, O'Grady, & Mariani, 2014; Selkoe & Toonen, 2006), making them ideal for resolving fine‐scale genetic structure, detecting recent changes in population demography and connectivity (Selkoe & Toonen, 2006), and inferring invasion routes (Mallez et al., 2013). We applied multiple data analysis methods including a new model‐based approach, approximate Bayesian computation (ABC). ABC has been successfully applied in invasion genetics studies (Estoup & Guillemaud, 2010; Guillemaud, Beaumont, Ciosi, Cornuet, & Estoup, 2010). It allows parameter estimation of complex evolutionary scenarios for which likelihoods are different or practically impossible to infer (e.g., Lombaert et al., 2011; Goss et al., 2014). Using these approaches, we aimed to (a) compare genetic diversity of *H. schachtii* populations from different continents; (b) assess their population structure and differentiation; and (c) compare evolutionary scenarios for the sampled populations using ABC.

## 2 MATERIALS AND METHODS

### 2.1 Sample collection and DNA preparation

From 2010 to 2015, we gathered a total of 231 individuals of *H. schachtii* from 12 populations in eight countries of Asia, Europe, North America, and Oceania (Table 1 and Figure 1). Cysts were sampled from roots of sugar beet and Chinese cabbage and then fixed in molecular‐grade pure ethanol (99.9%) until genomic DNA extraction. Specimens were identified based on morphology and then validated by sequence comparison of ITS1–5.8S‐ITS2 region among six *Heterodera* species (for details see “*Species identity of Heterodera schachtii”* in the Supporting information Appendix S1). As suggested by Plantard and Porte (2004), only one second‐stage larva per cyst was used to avoid genotyping closely allied individuals (in this species, larvae of the same cyst share at least the same mother). Total genomic DNA was extracted from single individuals using a lysis buffer containing 0.2 M NaCl, 0.2 M Tris‐HCl (pH 8.0), 1% (v/v) β‐mercaptoethanol, and 800 μg/ml proteinase‐K (Holterman et al., 2006), and then amplified using a whole genome amplification kit (GenomePlex Complete Whole Genome Amplification Kit; Sigma, St. Louis, MO, USA) to guarantee a sufficient quantity of DNA for subsequent microsatellite genotyping.

**Table 1:** Sampling information for the 12 *Heterodera schachtii* populations and genetic diversity indices of each population based on 15 microsatellite loci

No.	Abbreviation	Location	*N*	*H*_exp_	*N*o of alleles per pop	MLG	eMLG	Lambda
1	AU	Australia	10	0.422	38	10	10	0.900
2	GE	Germany	28	0.543	59	28	10	0.964
3	IR	Iran	10	0.459	38	10	10	0.900
4	KR_JS	Jeongseon, Korea	27	0.478	40	27	10	0.963
5	KR_SC	Samcheok, Korea	29	0.461	37	25	9.56	0.956
6	KR_TB	Taeback, Korea	38	0.479	65	38	10	0.974
7	NE	Netherlands	25	0.425	35	25	10	0.960
8	TU_AK	Aksaray, Turkey	10	0.503	36	10	10	0.900
9	TU_NI	Nigde, Turkey	10	0.420	31	10	10	0.900
10	UK	Ukraine	10	0.468	41	10	10	0.900
11	US_CA	California, USA	24	0.497	55	24	10	0.958
12	US_OR	Oregon, USA	10	0.429	37	10	10	0.900
	Total		231	0.614	42.6	226	9.99	0.995

Note

eMLG: expected number of MLGs at the lowest common sample size of 10; *H*_exp_: Nei's unbiased gene diversity; lambda: Simpson's diversity index; MLG: number of multilocus genotypes; *N*: number of individuals genotyped; No of alleles per pop: number of alleles per population.

### 2.2 PCR and genotyping

Fifteen polymorphic microsatellite markers developed by Kim et al. (2016) were amplified for each sample. The PCR conditions were as follows: each 20 μl reaction contained 13.3 μl of distilled water, 2 μl of 10× Ex Taq buffer (Mg2^+^ free), 0.8 μl of MgCl_2_, 1.6 μl of dNTP mixture, 0.8 μl of each primer (10 pmole), 0.2 μl TaKaRa Ex Taq (Takara Bio, Shiga, Japan), and 0.5 μl template DNA, with 1 cycle of initial denaturation at 95°C for 1 min, followed by 40 cycles of denaturation at 95°C for 30 s, annealing at 50–65°C for 30 s, and extension at 72°C for 30 s. The final elongation step was done at 72°C for 30 min. Negative controls (no template) were run in all PCR amplifications to check for contamination. PCR was repeated at least twice with modified conditions to confirm the results of reactions without target bands. Genotyping was performed using an ABI 3730 XL DNA Genetic Analyzer (Applied Biosystems, Foster City, CA, USA) and analyzed by GeneMapper 3.7 (see the genotyping data in the Supporting information Appendix S2).

### 2.3 Data analysis

#### 2.3.1 Genetic variation within and among populations

Hardy–Weinberg equilibrium (HWE) was examined for each locus using the function *hw.test ()* from the *pegas* package in *R* version 3.4.0 (R Core Team, 2016). We also tested HWE statistics for each locus at each population and then visualized the results as a heatmap in the package *lattice*. The percentage of missing data per locus and population was assessed using *info_table* in *poppr* 2.4.1 (Kamvar, Tabima, & Grünwald, 2014). Genetic diversity indices such as Nei's unbiased gene diversity (*H*_exp_), number of alleles per population, number of multilocus genotypes (MLG), expected number of MLGs (eMLG) at the lowest common sample size (=10 in this study), and Simpson's diversity index (Lambda) were calculated for each *H. schachtii* population using *poppr*. For each locus, the number of alleles, observed heterozygosity, expected heterozygosity, and evenness were also calculated.

Genetic differentiation between populations was estimated by calculating pairwise estimators of *F*_ST_ (Weir, 1996). The *ENA* (excluding null alleles) method, suggested by Chapuis and Estoup (2007), was applied to correct for the positive bias on *F*_ST_ statistics induced by the presence of null alleles. $F_{\text{ST}}^{\left[ENA\right]}$ values were quantified by the program FREENA (https://www1.montpellier.inra.fr/CBGP/software/FreeNA/) and then visualized as a heatmap in *R*.

To estimate the influence of the existence of null alleles on *H. schachtii*, we repeated analyses again, excluding the two loci that had a null allele frequency >0.19. This value is a threshold over which underestimation of expected heterozygosity due to null alleles becomes significant (Chapuis et al., 2008). The following phylogenetic and population structure analyses were also performed on both data sets (15 loci and 13 loci). For each analysis, multiple and model‐free approaches were used when possible to minimize the influence of null alleles.

#### 2.3.2 Phylogenetic analysis

Phylogenetic relationships were inferred at both the individual and population level. We calculated Provesti's distance (which can handle missing data) among all individuals in *poppr* with 10,000 bootstraps and used the distance matrix to create an unrooted neighbor‐joining (NJ) tree. A minimal spanning network (MSN) was built using Bruvo's distance (utilizing a stepwise mutation model) among all MLGs in the same package. Genetic distance within and between defined genotype groups was calculated and visualized as boxplots.

We then used POPULATION software v. 1.2 (Olivier Langella, CNRS UPR9034, France, https://bioinformatics.org/populations/) to generate a NJ tree among populations based on the chord distance (Cavalli‐Sforza & Edwards, 1967; *D_C_*) with 1,000 permutations. This genetic distance was selected because it is considered the most efficient way for generating an accurate phylogeny (Takezaki & Nei, 1996). FigTree version 1.4.3 (https://tree.bio.ed.ac.uk/software/figtree/) was used to view both NJ trees as radial dendrograms.

#### 2.3.3 Genetic cluster analysis

Genetic clusters within *H. schachtii* were first characterized with a Bayesian algorithm implemented in STRUCTURE version 2.3 (Pritchard, Stephens, & Donnelly, 2000). For each number of population clusters *K* (from 1–12), ten replicates were run using the admixture model, correlated allele frequencies and the prior population information with 100,000 Markov chain Monte Carlo (MCMC) iterations after a burn‐in of 10,000 steps. Structure Harvester version 0.6.92 (Earl & vonHoldt, 2012) was applied to visualize the STRUCTURE output and to choose the optimal value of *K* based on the Delta *K* method (Evanno, Regnaut, & Goudet, 2005). The 10 independent replicates for each *K* were merged using CLUMPP version 1.1.2 (Jakobsson & Rosenberg, 2007), and the final plots were generated using DISTRUCT version 1.1 (Rosenberg, 2004). We further carried out a discriminant analysis of principal components (DAPC) in the *R* package *adegenet* version 2.0.1 (Jombart, 2008) to validate results obtained from STRUCTURE. DAPC is a multivariate and model‐free approach for finding the principal components that explain the most among‐group variation while minimizing within‐group variation (Jombart, Devillard, & Balloux, 2010). To investigate possible hierarchical structuring, additional analyses were performed for each method after excluding Korean populations. Finally, a hierarchical analysis of molecular variance (AMOVA) (Excoffier, Smouse, & Quattro, 1992) was conducted based on 10,000 permutations in ARLEQUIN 3.5 (Excoffier & Lischer, 2010) to calculate the partitioning of genetic variation based on the grouping suggested above.

#### 2.3.4 Evolutionary scenarios estimated using ABC

Competing evolutionary scenarios among *H. schachtii* population were compared using an ABC approach in DIYABC version 2.1.0 (Cornuet et al., 2014). Considering little information is available about the international origin and dispersal of *H. schachtii*, reconstructing evolutionary relationships among all sampled populations is unrealistic, and so in our analysis, we focused on the putative origins of the newly documented Korean populations. To simplify scenario comparison, we applied a two‐step procedure. The first step was to reconstruct a basic evolutionary scenario without the Korean populations. Three pooled populations were considered in this step, based on the three STRUCTURE‐defined groups: NE, TU (TU_AK and TU_NI), and CORE (UK, US_CA, US_OR, AU, GE, and IR). Pooling populations with shared history is an efficient way to reduce the complexity of candidate scenarios and has been used in numerous other studies (see Pedreschi et al., 2014; Jeffries et al., 2017). Six evolutionary scenarios that considered population history events including divergence, admixture, and population size variation were evaluated. The CORE population was assumed to be the ancestral population in each scenario considering its broad distribution among continents. The second step was to reconstruct the origin of the Korean population based on the scenario with the highest support value in the first step. Four scenarios were compared without considering mixture events, because the Korean populations were found to be monophyletic. To integrate the effects of an unsampled source population on DIYABC inferences (see Lombaert et al., 2011), a ghost population was assumed in scenario 2–4 to represent an unsampled source population for the Korean group.

For both steps, one million simulated data sets were run for each scenario to build a reference table under a generalized stepwise mutation model with mean mutation rate ranging from 1.00E‐004 to 1.00E‐3 and uniform prior distribution. We measured the similarity between real and simulated data sets using summary statistics both within and between populations as suggested in the DIYABC manual, including the mean number of alleles, mean genetic diversity and mean size variance for single population, and pairwise *F*_ST_, classification index, and (dμ)^2^ distance for pairs of populations. A pre‐evaluation step based on a principal components analysis (PCA) was performed to ensure that at least one combination of scenarios and priors can produce simulated data sets that are close enough to the observed data set.

The relative posterior probabilities of the competing scenarios were estimated by a logistic regression analyses on the 1% of simulated data sets closest to the observed data set (Cornuet, Ravigné, & Estoup, 2010). The model with the highest posterior probability and for which the 95% confidence interval (CI) did not overlap with the CIs of other models was considered the best model. A model checking computation was then applied to evaluate the goodness of fit of the most highly supported scenario using PCA, in which the observations were the simulated data sets and the variables were the summary statistics including all single‐population and two‐population statistics. The “Confidence in scenario choice” function was used to estimate the proportion of type 1 and type 2 errors based on 1,000 pseudo‐observed data sets. Small type II errors provide good confidence in the selected scenario even if the type I errors are large (Bermond et al., 2012).

## 3 RESULTS

### 3.1 Null alleles and their influence on results

Null alleles were prevalent among populations, especially in Iran (0.200) and two Turkey populations (0.373 and 0.400 for TU_AK and TU_NI, respectively) (Supporting information Figure S1 in Appendix S1). The mean null allele frequency over all populations and loci was 0.100, with frequencies averaged over loci ranging from 0.013 to 0.251. DNA samples that failed to yield products at some loci could nevertheless be amplified successfully at other loci, a pattern implying the existence of null alleles for most loci and populations (Chapuis et al., 2008). Deviation from HWE was observed for each locus (Supporting information Table S1) and also for most statistics of each locus in each population (Supporting information Figure S2 in Appendix S1). Two loci were observed with null allele frequencies over 0.19 (HS005 and HS033), and so analyses were repeated excluding them. Since the analyses using all 15 loci and yielded similar results (in genetic diversity, phylogeny, and population structure) as the analyses using 13 loci, the following results are presented based on all 15 loci. Results using 13 loci are provided in the Supporting information Appendix S1.

### 3.2 Genetic variation within and among populations

All fifteen loci were highly polymorphic but had different degrees of diversity, varying from 5 alleles in both HS021 and HS033 to 14 alleles in HS037, with a mean value of 7.73 across all loci (Table 2). For each locus, the observed and expected heterozygosity ranged from 0.12 (HS034) to 0.97 (HS009) and from 0.23 (HS034) to 0.82 (HS008), respectively, and the evenness varied from 0.38 (HS034) to 0.89 (HS021). For each population, Nei's unbiased gene diversity ranged from 0.422 in AU to 0.543 in GE, with a global value of 0.614. The number of alleles per population ranged from 31 in TU_NI to 65 in KR_TB, with an average of 42.6 (Table 1). The genotype accumulation curve reached a plateau by 15 loci (Supporting information Figure S3 in Appendix S1), showing ample power to discriminate among individuals. A total of 226 MLGs were discovered, varying for each population from 10 in six populations to 38 in KR_TB. The eMLG for each population was always equal to the lowest common sample size (10) except for KR_SC (9.56). Simpson's index (lambda) was always above 0.900 with the highest value (0.974) in KR_TB (Table 1).

**Table 2:** Genetic diversity detected at 15 microsatellite loci in 231 individuals of *Heterodera schachtii*

locus	*N_A_*	% missing	*H*_O_	*H*_E_	Evenness
HS005	9	24.24	0.23	0.49	0.50
HS006	6	4.76	0.80	0.66	0.87
HS008	10	7.79	0.87	0.82	0.86
HS009	8	4.33	0.97	0.63	0.84
HS011	7	3.03	0.88	0.73	0.73
HS016	6	1.3	0.96	0.63	0.88
HS021	5	9.52	0.93	0.63	0.89
HS025	7	16.02	0.62	0.54	0.70
HS028	11	6.93	0.33	0.76	0.67
HS030	7	14.72	0.69	0.73	0.80
HS033	5	25.11	0.03	0.52	0.75
HS034	8	9.52	0.12	0.23	0.38
HS035	6	7.79	0.38	0.58	0.66
HS036	7	2.6	0.61	0.57	0.59
HS037	14	12.55	0.16	0.67	0.52
Mean	7.73	10.01	0.23	0.61	

Note

% missing: percentage of missing data at each locus; Evenness: the distribution of genotype abundances; *H*_E_: expected heterozygosity; *H*_O_: observed heterozygosity; *N*_A_: total number of observed alleles

**Figure 2:**
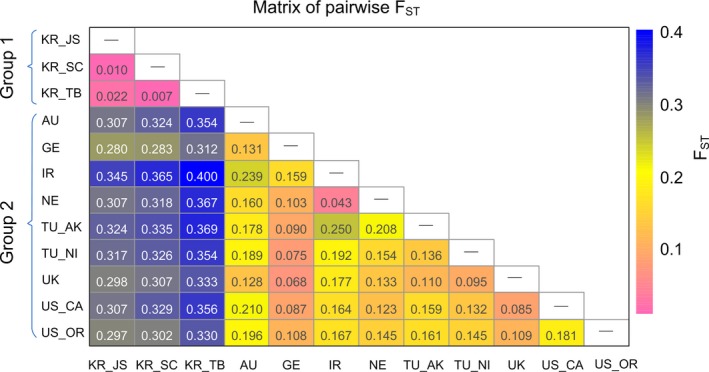
Heatmap visualizing pairwise $F_{\text{ST}}^{\left[ENA\right]}$ values between the *Heterodera schachtii* populations

According to Hartl and Clark (1997)’s criteria, the overall level of population differentiation was great, with global $F_{\text{ST}}^{\left[ENA\right]}$ = 0.235 (95% CI: 0.185–0.293). The three Korean populations (hereafter referred to as group 1) showed very great differentiation from the other nine populations (hereafter referred to as group 2), with large $F_{\text{ST}}^{\left[ENA\right]}$ values ranging from 0.280 to 0.400 (Figure 2). However, substantial genetic homogeneity was observed among Korean populations, with all $F_{\text{ST}}^{\left[ENA\right]}$ values below 0.05. Population differentiation among group 2 varied from little (e.g., between IR and NE, $F_{\text{ST}}^{\left[ENA\right]}$ = 0.043) to great (between IR and TU_AK, $F_{\text{ST}}^{\left[ENA\right]}$ = 0.250), with most comparisons displaying moderate differentiation (Figure 2).

### 3.3 Phylogenetic relationships

**Figure 3:**
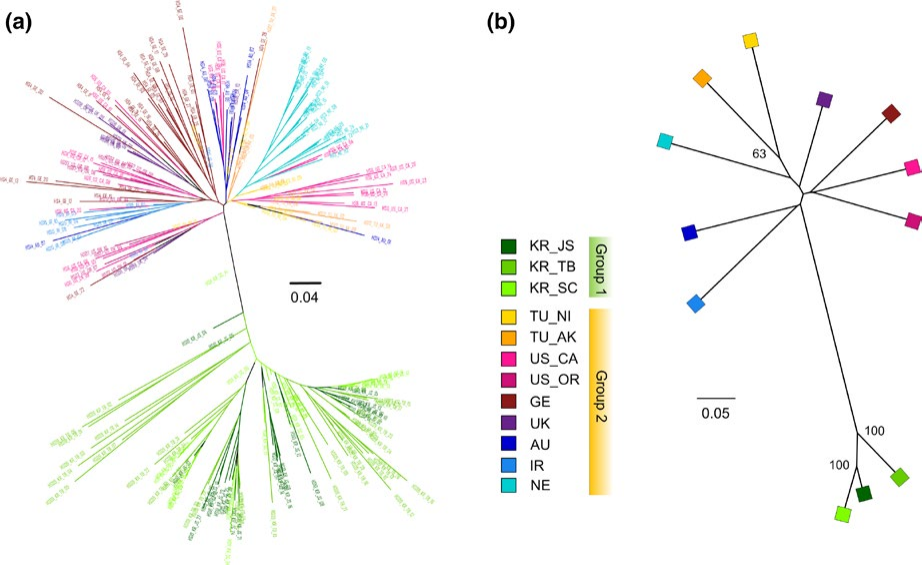
Phylogenetic trees displaying the relationship (a) among all *Heterodera schachtii* individuals based on Provesti's distance and (b) among all populations based on *D*_C_ distance

**Figure 4:**
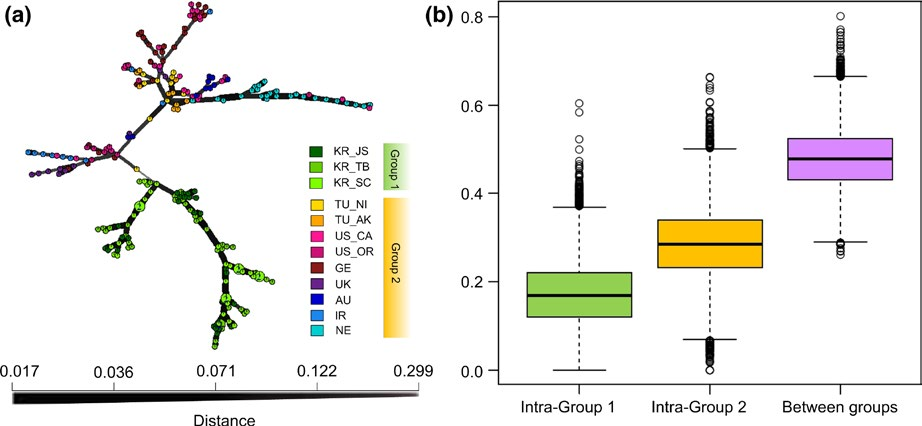
(a) Minimal spanning network displaying the topology among all multilocus genotypes (MLGs) of 15 microsatellite loci based on Bruvo's distance; (b) boxplots of Bruvo's distances (*y*‐axis) among MLGs of groups 1 (intra‐group 1) and 2 (intra‐group 2) and between MLGs of groups 1 and 2 (between groups)

The individual NJ tree (Figure 3a) revealed two divergent clusters with a clear geographic pattern, corresponding to groups 1 and 2 defined above. No subgroups were discovered among three Korean populations in group 1, while for group 2, most individuals in NE and IR assembled closely together as a subclade. The population NJ tree also revealed a clear distinction between Korean populations and all non‐Korean populations, with a long branch connecting these two clades (Figure 3b). The MSN generated similar results, as two divergent lineages appeared which separated Korean MLGs from the rest (the thin line between groups represents large genetic distance in Figure 4a). The median genetic distance among MLGs of groups 1 and 2 was 0.168 and 0.284, respectively, while the distance between them was up to 0.478 (Figure 4b).

### 3.4 Population structure

**Figure 5:**
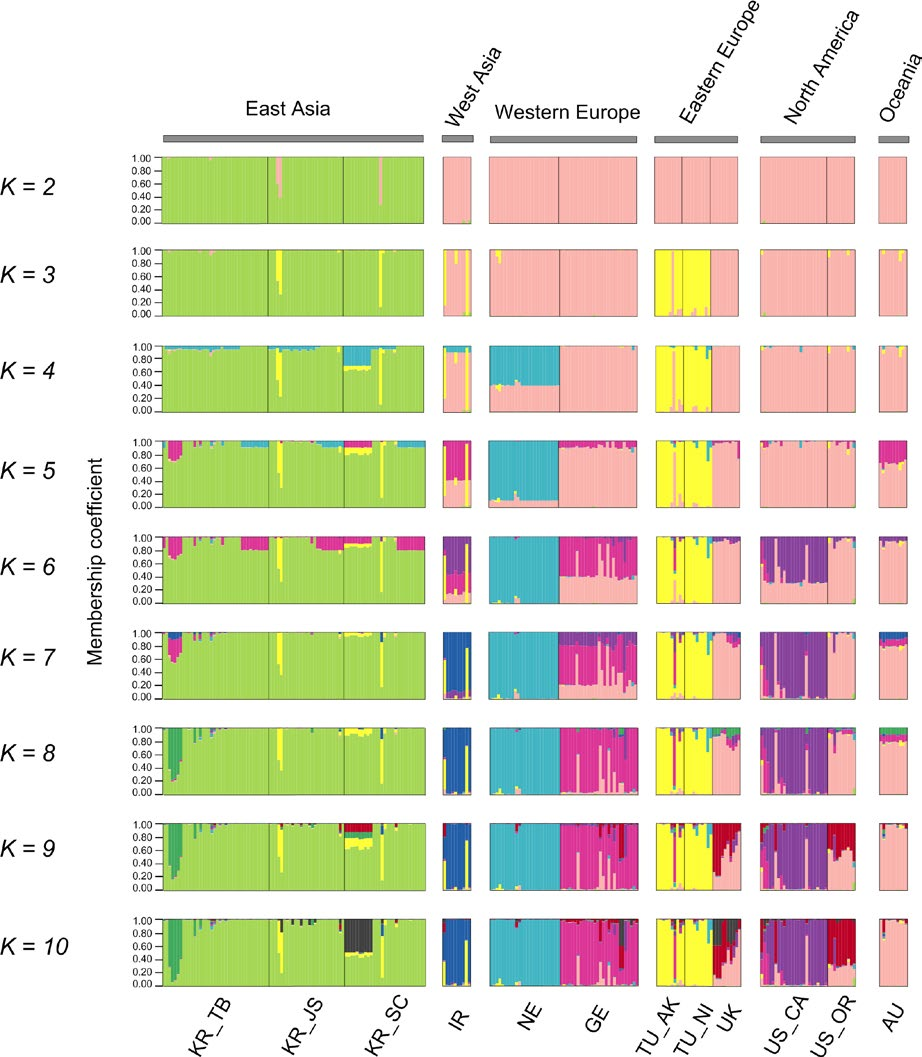
Clustering analysis of *Heterodera schachtii* genotypes with admixture proportions for *K* from 2 to 10 in STRUCTURE. Each sample is indicated by a narrow bar divided into *K* colored segments showing the individual's mean membership coefficient for each of the clusters *K*. Population locations and the continent they belong to are also indicated at the bottom and top of the bar plots, respectively

For the STRUCTURE analysis, Δ*K* indicated the best grouping occurred at *K* = 2 (Supporting information Figure S4a in Appendix S1), which suggests the three Korean populations (green cluster) had a distinct genetic composition from all other populations (pink cluster) (Figure 5). With increasing *K*, additional clusters continuously appeared which suggested there was significant substructure among the nine populations. For example, when *K* = 3, two Turkey populations (TU_AK and TU_NI) separated from the other populations; and when *K* = 4, NE became separated; this trend continued until *K* = 10 (Figure 5). This pattern was also supported by the STRUCTURE analysis in which only the nine populations in group 2 were considered, since the highest Δ*K*′ was obtained when *K* = 3 (Supporting information Figure S4b,c in Appendix S1).

**Figure 6:**
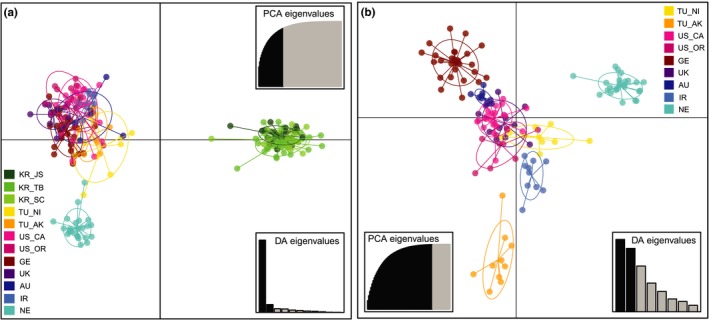
Population clusters based on discriminant analysis of principal components along with discriminant analysis (DA) eigenvalues and principal components analysis (PCA) eigenvalues retained for *Heterodera schachtii* individuals from (a) all 12 populations and (b) the nine populations when Korean populations are excluded

The DAPC plots illustrated a similar pattern as the STRUCTURE results. When all populations were included, we retained 30 principal components (PCs) that explained 89.5% of the total variance. Two completely separate genetic clusters appeared, corresponding to groups 1 and 2 (Figure 6a). The first two DA eigenvalues were 995.1 and 108.1, respectively. When only group 2 was analyzed, several subgroups appeared, such as NE, TU_AK, and AU, indicating the presence of substructure within group 2 (Figure 6b). The first 60 PCs explained 99.6% of the total variance.

Based on the population clustering suggested above (groups 1 and 2), AMOVA analysis revealed that most genetic variation came from variation within populations (73.84%, *p < *0.0001), followed by variation among groups (23.02%, *p* = 0.0045) and variation among populations within groups (3.13%, *p* < 0.0001) (Table 3).

**Table 3:** Analysis of molecular variance (AMOVA) for *Heterodera schachtii* samples based on groups 1 and 2

Source of variation	*df*	Sum of squares	Variance components	Percentage of variation	Fixation indices	*p*‐value
Among groups	1	92.468	0.3961	23.02	*F*_CT_ = 0.2303	0.0045
Among populations within groups	10	32.112	0.0539	3.13	*F*_SC_ = 0.0407	<0.0001
Within populations	450	571.595	1.2702	73.84	*F*_ST_ = 0.2616	<0.0001

### 3.5 Evolutionary scenario reconstruction

**Figure 7:**
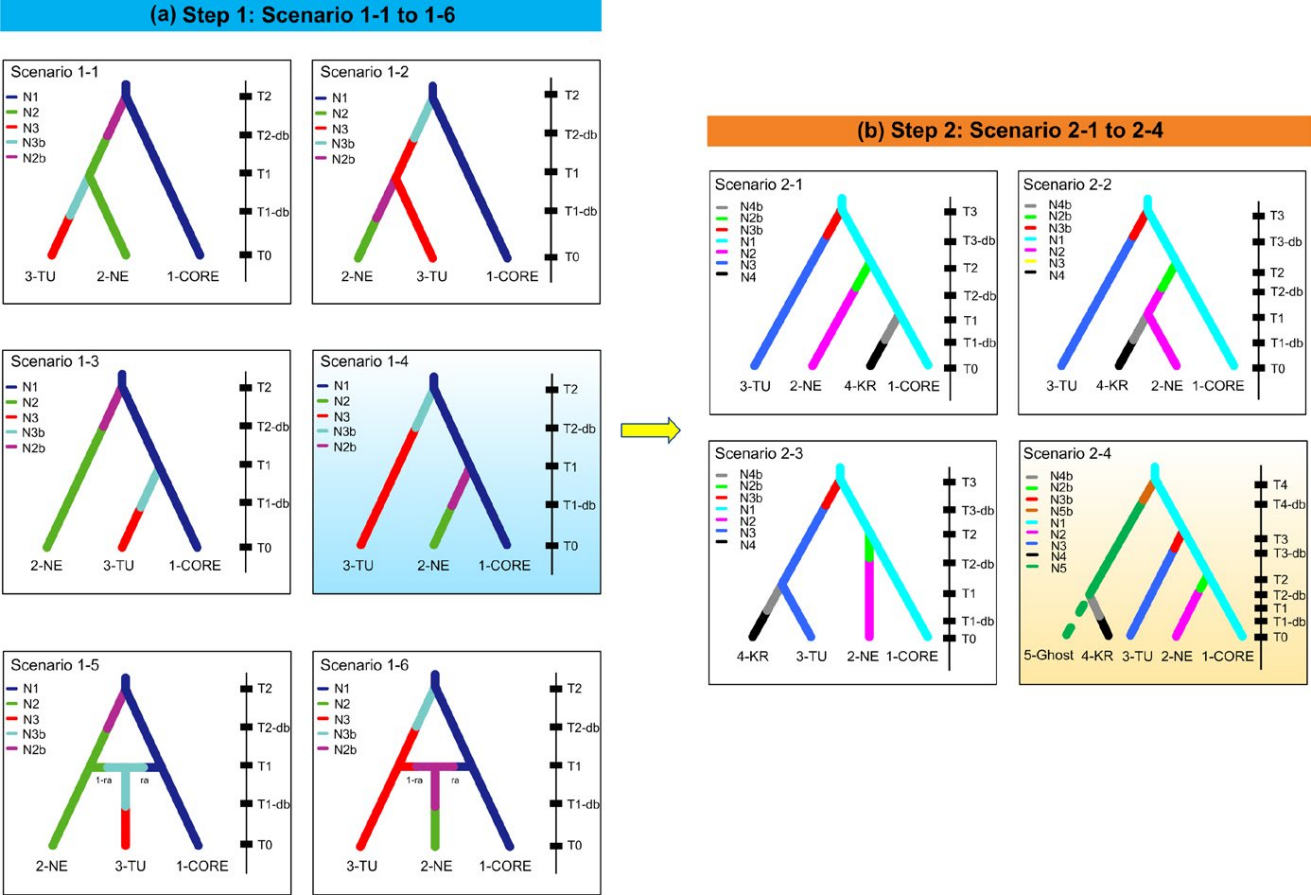
Evolutionary scenarios for *Heterodera schachtii* expansion tested by the approximate Bayesian computation (ABC) approach. (a) Step 1: comparison of scenarios 1–1 to 1–6 when the CORE, NE, and TU are considered; (b) Step 2: based on the best‐supported model in step 1, comparison of scenarios 2–1 to 2–4 to test the origin of Korean populations (KR). The best‐supported model in each step is highlighted in blue and orange, respectively. Scenarios are shown together with their respective phylogenetic tree and relative times of divergence (0 indicates the present day and *t* on top of the bar indicates the oldest split; BD: the duration of the bottleneck period during introduction; note that times are not to scale)

The model pre‐evaluation using PCA illustrated that the observed data fell within the cluster of simulated data points based on the scenarios in each step (Supporting information Figure S5a,b in Appendix S1), indicating the appropriateness of pursuing comparative analyses. In step one, model comparisons identified Scenario 1–4 as the most probable scenario with the highest posterior probability (PP) of 0.663 (95% CI: 0.642–0.684) based on logistic regression (Supporting information Figure S5c in Appendix S1). The next most highly supported scenario was Scenario 1–6 (PP = 0.250), and its 95% CI (0.230–0.269) had no overlap with Scenario 1–4. Under Scenario 1–4, TU and NE populations successively originated from the CORE population, each passing through a bottleneck stage (Figure 7a). In the assessment of confidence in scenario choice, the type I error was 0.311 and the mean type II error was 0.097 (ranging from 0.046 to 0.227). This result was corroborated by the PCA, in which the observed data set was closely matched by many simulated data points from the posterior sample of Scenario 1–4, indicating that the simulations produced data sets similar to the observed data (Supporting information Figure S5e in Appendix S1).

In step two, Scenario 2–4 was the most highly supported scenario, with PP = 0.986 (CI: 0.976–0.996) and no overlap with the other scenarios (Supporting information Figure S5d in Appendix S1). In this scenario, a ghost population split with the CORE population early in time, the TU and NE populations were later derived from the CORE separately (Figure 7b), and the Korean population originated from the ghost population recently. A bottleneck effect is assumed for each colonizing event. In the “confidence in scenario choice” analysis, the type I error was 0.225 and the mean type II error was 0.112 (min = 0.110 and max = 0.133), showing high statistical power for distinguishing between the alternative scenarios. In the PCA simulations, the observed data closely resembled the posterior sample of Scenario 2–4 (Supporting information Figure S5f in Appendix S1).

## 4 DISCUSSION

Biological invasion represents an important area of research in global change biology. About half a million species have been translocated due to anthropogenic activities, causing far‐reaching ecological and economic impacts around the world (Pimentel et al., 2001). Plant‐parasitic nematodes, despite their capacity for huge economic damage, are still overlooked in evolutionary studies, and there is little information about their invasion biology (Wielgoss, Taraschewski, Meyer, & Wirth, 2008). In this study, we assessed the phylogeographic patterns of the highly invasive nematode *H. schachtii* from four continents using 15 polymorphic microsatellites. Unique features with regard to null alleles were observed in the data set, and unexpected patterns of population diversity and structure, as well as the most likely evolutionary scenarios regarding the origin of Korean populations, were inferred using multiple approaches.

### 4.1 High prevalence of null alleles

The microsatellite loci of *H. schachtii* in this study are highly prone to null alleles, with a mean frequency up to 10%. Null alleles are alleles that fail to be genotyped due to mutations on primer‐binding sites (Brookfield, 1996) or suboptimal PCR conditions (Dakin & Avise, 2004). They are pervasive in microsatellite makers and have been reported in a wide range of taxa (e.g., Kokita, Takahashi, & Kumada, 2013; Karn & Jasieniuk, 2017). The existence of null alleles can obscure the true relationships between different genotypes and cause serious problems in parentage analysis (Huang et al., 2016). However, its impact on phylogeographic analysis has not been fully evaluated.

Chapuis et al. (2008) used computer simulations to comprehensively appraise the effect of null alleles on various analyses. Their results showed that null alleles cause an underestimation of diversity estimations and an overestimation of population differentiation. They also demonstrated that null alleles have a limited effect on NJ phylogenetic trees based on *D*_C_ genetic distances. In this study, our analyses of *F*_ST_ and *D*_C_ distance based on all loci were consistent with the conclusions of Chapuis et al. (2008): The *F*_ST_ including null alleles (INA) showed a higher value than the *F*_ST_ excluding null alleles ($F_{\text{ST}}^{\left[ENA\right]}$ = 0.244 [95% CI: 0.188–0.307] versus $F_{\text{ST}}^{\left[ENA\right]}$ = 0.235 [95% CI: 0.185–0.293]); and the *D_C_* distance heatmaps both with and without the “INA correction” (following Chapuis & Estoup, 2007) (Supporting information Figure S6a,b in Appendix S1) showed similar values.

We also performed the analyses after excluding the two loci whose null allele frequency was higher than 0.19. The expected heterozygosity values based on just 13 loci were generally lower than the values using all 15 loci, both for individual and overall populations (Supporting information Table S2). In the phylogenetic and grouping analyses using 13 loci, all the results including the individual and population NJ trees (Supporting information Figure S7 in Appendix S1), MSN tree (Supporting information Figure S8 in Appendix S1), and DAPC clustering (Supporting information Figure S9 in Appendix S1) were similar to the analyses based on 15 loci. This indicates that phylogeographic patterns in this study are robust and not significantly biased by the high frequency of null alleles.

### 4.2 Deep genetic divergence

The most striking pattern uncovered in this study is the high divergence of the three Korean populations (group 1) from the other populations worldwide (group 2) in the phylogenetic and genetic clustering analyses. Large genetic distance was estimated between groups 1 and 2: The median Bruvo's distance of MLGs between groups was nearly 3‐ and 1.5‐fold larger than the intra‐group distances (Figure 4b). All these lines of evidence indicated that the Korean populations represent a cryptic lineage of *H. schachtii*. Although many previous studies using various approaches have been conducted on this species (e.g., Plantard & Porte, 2004; Madani et al., 2007), no cryptic lineages among *H. schachtii* populations have yet been reported, probably due to insufficient sampling coverage or the low resolution of the genetic markers applied in earlier studies. Cryptic PPN species are probably common, though difficult to detect using morphology alone (Palomares‐Rius, Cantalapiedra‐Navarrete, & Castillo, 2014). In recent years, with the integration of new molecular tools into taxonomy, cryptic species and lineages of PPNs have been frequently reported (reviewed by Jones et al., 2013).

### 4.3 Hierarchical population subdivision

Besides the significant difference between Korean and non‐Korean populations, population substructure was also observed among the populations within group 2. Great genetic differentiation [$F_{\text{ST}}^{\left[ENA\right]}$ > 0.15] according to Hartl and Clark (1997)] was observed for 17 out of 36 pairwise comparisons (Figure 2). The STRUCTURE and DAPC results supported the separation of TU and NE populations from each other and the other populations. The other two European populations, GE and UK, were closely associated with the group 2 populations found outside of Europe. However, further population subdivision was also inferred with increasing *K* in the STRUCTURE analysis.

Passive dispersal of cysts by human activities, water, or wind is assumed to enhance PPN gene flow among distant regions (Gavassoni, Tylka, & Munkvold, 2001; Kwon et al., 2016), but very few studies actually address global‐scale genetic patterns in PPNs. The various degrees of genetic differentiation we report above indicate hierarchical population subdivision among different *H. schachtii* populations. This result challenges the conclusions of previous studies on the population structure of *H. schachtii* and other nematodes; for instance, Plantard and Porte (2004) found weak genetic differentiation among *H. schachtii* populations (~175 km apart) in northern France based on five microsatellite loci, and one study of *Caenorhabditis elegans*, a free‐living soil nematode, also found weak differentiation between populations in Australia, Germany, and the United States (Koch, Luenen, Horst, Thijssen, & Plasterk, 2000). We suggest that for *H. schachtii*, human‐mediated transport may have played an important role in its broad dispersal among different continents (Subbotin et al., 2010). Bottleneck events likely occurred in each invasive population, resulting in genetic differentiation between source and introduced populations (Wu et al., 2009). The hierarchical population subdivision of *H. schachtii* may be attributed to different divergence times between invasive and source populations, since populations that broke off earlier would have had more time to accumulate mutations (Estoup & Guillemaud, 2010).

### 4.4 Evolutionary scenario reconstruction

*H. schachtii* is considered native to Europe with observational data from most beet‐farming areas, but it has now attained a global distribution (Cooke, 1992). Our group 2 displays a wide distribution across West Asia, Europe, North America, and Oceania (Figure 5). However, the Korean populations surprisingly did not cluster with any of these populations, and show no evidence of having been introduced from these regions, suggesting the *H. schachtii* lineage that so successfully expanded out of Europe and around the globe is not related to the recent Korean invasion. Evolutionary scenario comparison indicates that a ghost population diverged early from the CORE population, accumulated large genetic differences, and subsequently was the source of the Korean invasion. Considering the substantial homogeneity among the Korean populations, they probably originated from the ghost population during a single introduction event and then subsequently spread in the Korean Peninsula. However, due to *H. schachtii*'s wide distribution and the lack of precise knowledge of the relationships and historical movements among the studied populations, it is not currently feasible to pinpoint the location of the ghost population. Note that to simplify possible candidate scenarios, we did not resolve relationships among the CORE populations. In fact, the populations of the CORE have relatively low levels of differentiation, making it unsafe to reconstruct a “true” population tree to estimate their introduction routes (Estoup & Guillemaud, 2010). Additional sampling will be necessary to better characterize the origin of the Korean lineage and elucidate the finer details of global evolutionary patterns in *H. schachtii*. Nevertheless, this study provides an important phylogeographic perspective on *H. schachtii* that can help to monitor its spread and track further invasion events in East Asia.

## 5 CONCLUSION

Biological invasions and their underlying mechanisms have become increasingly better understood by the application of an evolutionary perspective (Chown et al., 2015; Lee, 2002; Lee & Gelembiuk, 2008). However, current research on nematode invasions proceeds slowly, with few studies focusing on intraspecific diversity, especially on a global scale (Wielgoss et al., 2008). Understanding the genetic patterns of PPNs and reconstructing their evolutionary scenarios are timely problems. Based on polymorphic microsatellites and multiple analytical approaches (e.g., the model‐based ABC approach), we assessed the genetic differentiation and structure of the highly invasive nematode *H. schachtii* from four continents and discussed the most likely evolutionary scenarios behind these changes. These data are an essential foundation for creating strategies for better controlling and preventing the spread of *H. schachtii* and also represent a good model system for studying important questions in the invasion genetics of nematodes (Subbotin et al., 2010).

## 6 DATA ARCHIVING STATEMENT

Genbank accessions of the fifteen microsatellite loci used in this study are KT716061‐KT716075, and the microsatellite genotyping data for *Heterodera schachtii* are available in the Supporting information Appendix S2.

## CONFLICT OF INTEREST

The authors declare no competing commercial interests.
