# Supplementary material for: Phylogeography of the highly invasive sugar beet nematode, Heterodera schachtii (Schmidt, 1871), based on microsatellites
Source: Evol Appl. 2018 Oct 24;12(2):324–36. doi: 10.1111/eva.12719 (PMC6346664; doi:10.1111/eva.12719)
Supplement: Supplementary file 1 [file EVA-12-324-s001.docx]

**Supporting information:**

**Table S1** Hardy-Weinberg equilibrium (HWE) test for each locus using the function *hw.test()* from the *pegas* package.

| Locus | chi^2 | df | Pr(chi^2 >) | Pr.exact |
| --- | --- | --- | --- | --- |
| HS005 | 258.94972 | 36 | 0.00E+00 | 0 |
| HS006 | 119.21662 | 15 | 0.00E+00 | 0 |
| HS008 | 160.17424 | 45 | 7.99E-15 | 0 |
| HS009 | 643.11582 | 28 | 0.00E+00 | 0 |
| HS011 | 140.88557 | 21 | 0.00E+00 | 0 |
| HS016 | 204.99198 | 15 | 0.00E+00 | 0 |
| HS021 | 161.04437 | 10 | 0.00E+00 | 0 |
| HS025 | 182.26262 | 21 | 0.00E+00 | 0 |
| HS028 | 869.15299 | 55 | 0.00E+00 | 0 |
| HS030 | 141.43459 | 21 | 0.00E+00 | 0 |
| HS033 | 382.97795 | 10 | 0.00E+00 | 0 |
| HS034 | 444.49518 | 28 | 0.00E+00 | 0 |
| HS035 | 220.37622 | 15 | 0.00E+00 | 0 |
| HS036 | 38.07993 | 21 | 1.26E-02 | 0 |
| HS037 | 1265.12346 | 91 | 0.00E+00 | 0 |

**Table S2** Genetic diversity indices of each population based on 13 microsatellite loci

|  | Population | Hexp | N. alleles per pop | MLG | eMLG | lambda |
| --- | --- | --- | --- | --- | --- | --- |
| 1 | AU | 0.487 | 36 | 10 | 10 | 0.900 |
| 2 | GE | 0.548 | 50 | 28 | 10 | 0.964 |
| 3 | IR | 0.420 | 30 | 10 | 10 | 0.900 |
| 4 | KR_JS | 0.472 | 35 | 27 | 10 | 0.963 |
| 5 | KR_SC | 0.473 | 32 | 25 | 9.56 | 0.956 |
| 6 | KR_TB | 0.484 | 57 | 37 | 9.94 | 0.972 |
| 7 | NE | 0.447 | 31 | 24 | 9.85 | 0.957 |
| 8 | TU_AK | 0.545 | 35 | 10 | 10 | 0.900 |
| 9 | TU_NI | 0.459 | 28 | 10 | 10 | 0.900 |
| 10 | UK | 0.479 | 35 | 10 | 10 | 0.900 |
| 11 | US_CA | 0.547 | 52 | 24 | 10 | 0.958 |
| 12 | US_OR | 0.446 | 35 | 10 | 10 | 0.900 |
|  | Total | 0.631 | 38 | 223 | 9.99 | 0.995 |

*H_exp_*, Nei’s unbiased gene diversity; N. alleles per pop, number of alleles per population; MLG: number of multilocus genotypes; eMLG: expected number of MLGs at the lowest common sample size of 10; lambda: Simpson’s diversity index.


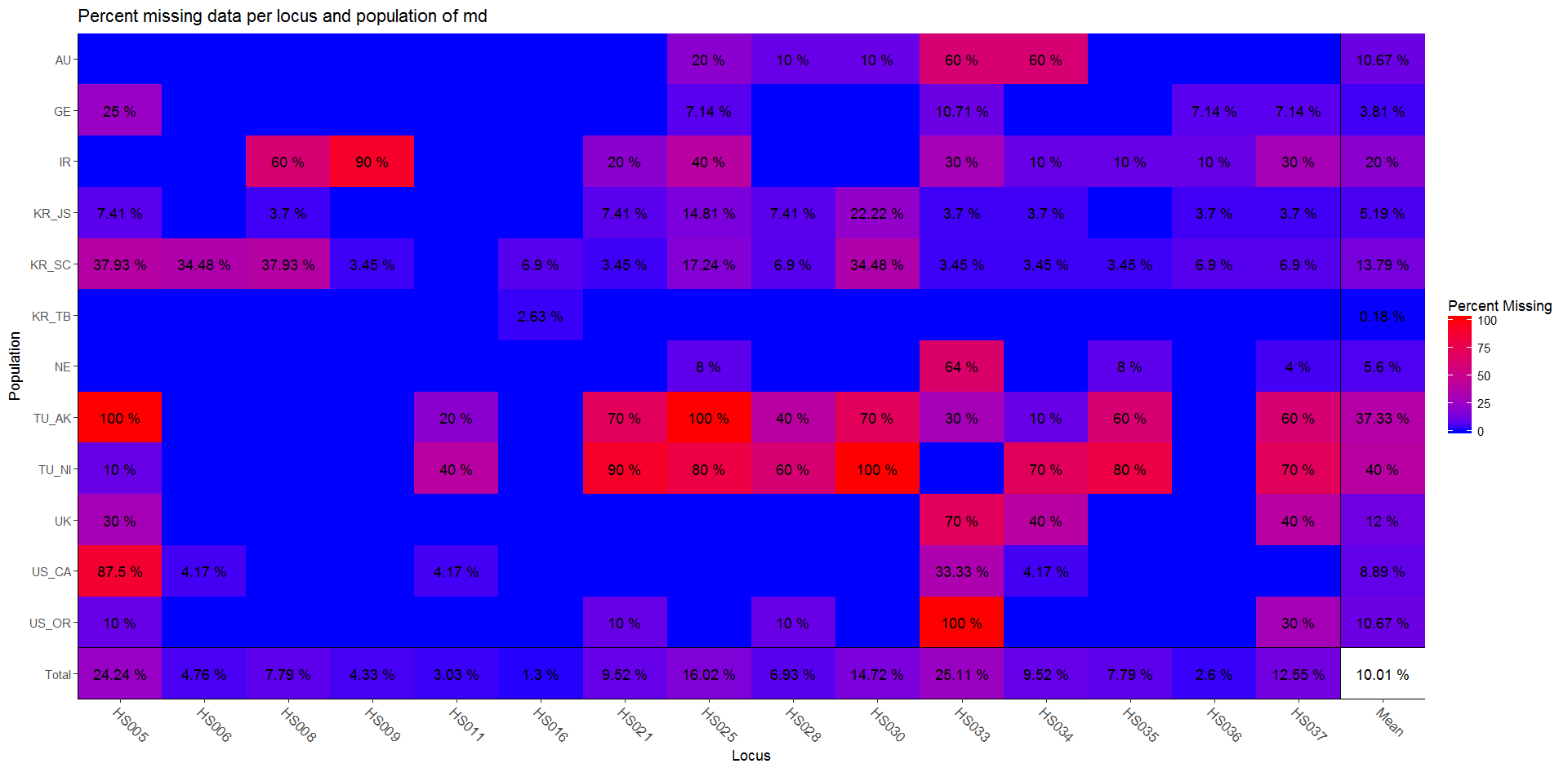


**Fig. S1** Percent missing data per locus and population of *Heterodera schachtii*.


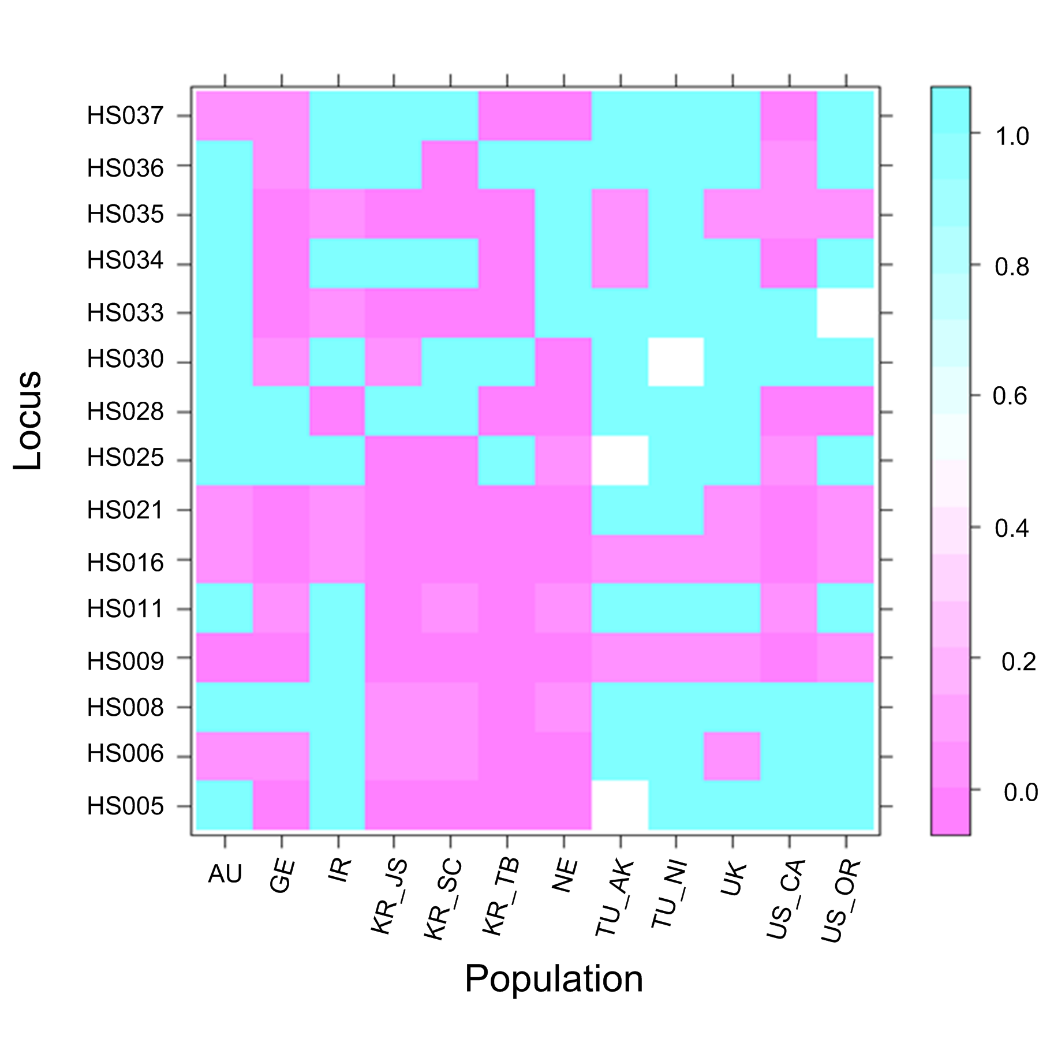


**Fig. S2** Heatmap showing significant departures of each locus per population from HWE. All loci shown in pink are loci that are not under HWE with p ≤ 0.05.


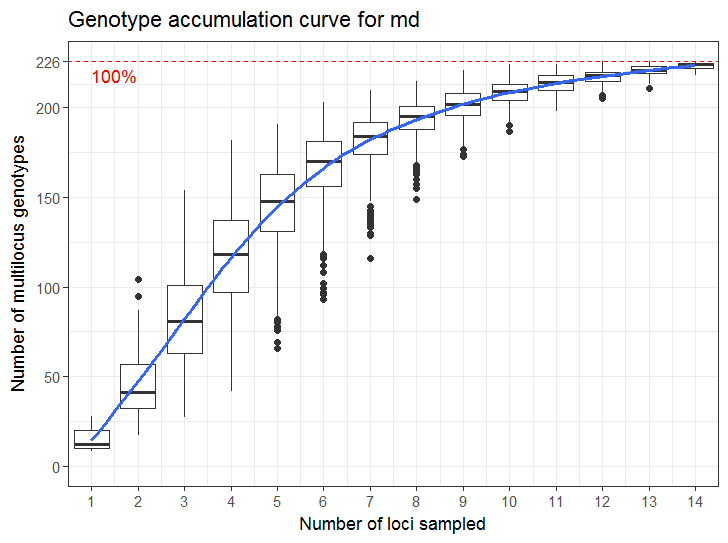


**Fig. S3**. Genotype accumulation curve over all 15 microsatellite loci.


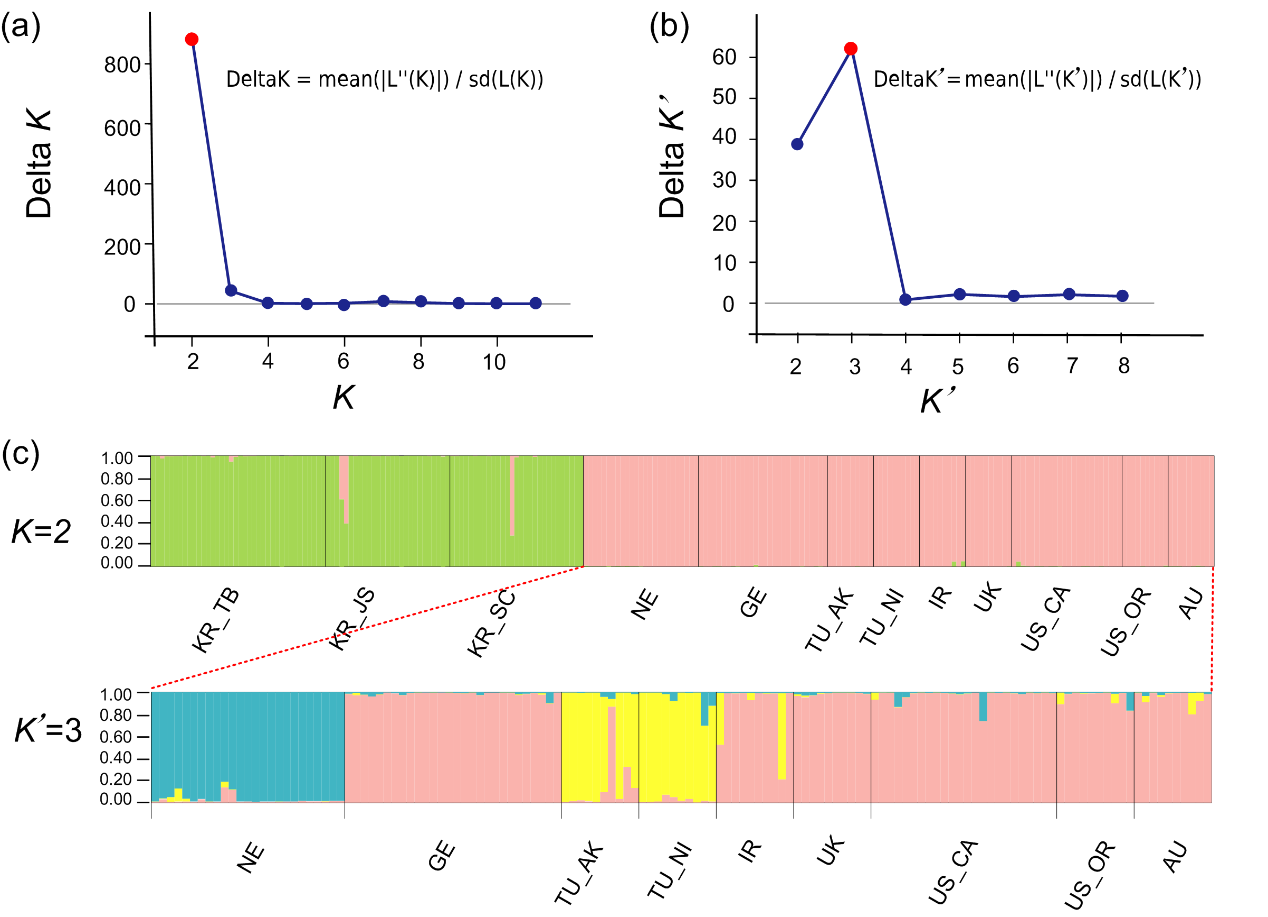


**Fig. S4** (a) Estimating the true number of clusters (*K*) with Δ*K* for all *Heterodera schachtii* populations; (b) Estimating the true number of clusters (*K’*) with Δ*K*’ for the nine populations when the Korean populations are excluded; (c) Based on the highest *K* (= 2) and *K’* (= 3) selected in (a) and (b), the clustering results for *H. schachtii* individuals from 12 populations and 9 populations (Korean populations excluded) in STRUCTURE.


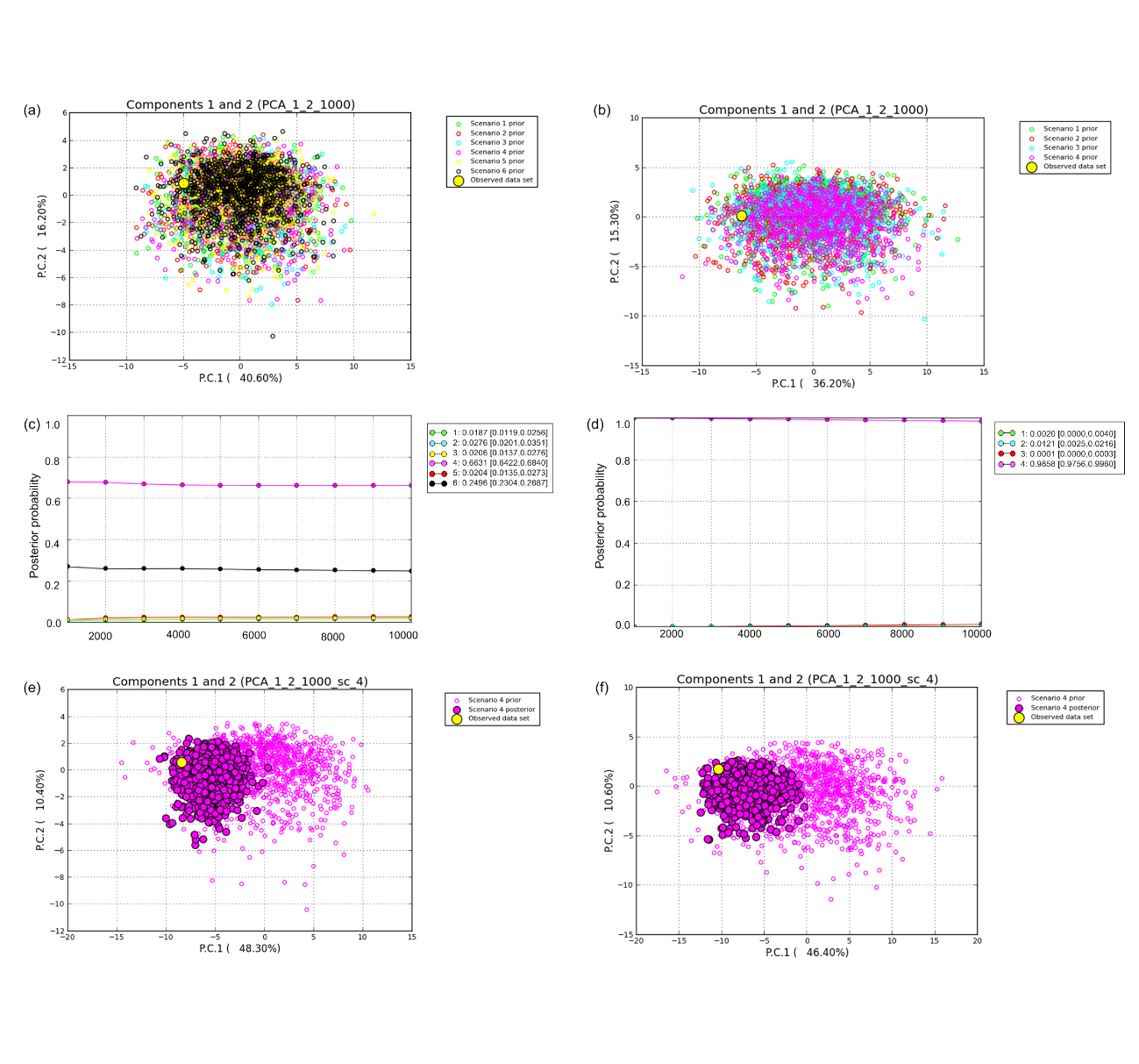


**Fig. S5** Results of the DIYABC analyses for both steps (a, c, e for step 1; b, d, e for step 2). (a), (b): validation of prior choice; (c), (d): model comparison based on a logistic regression approach, with the key showing the posterior probabilities [95% confidence intervals] for each scenario; (e), (f): posterior predictive check of model fit for the most highly supported scenario in each step (scenario 1-4 and 2-4, respectively).


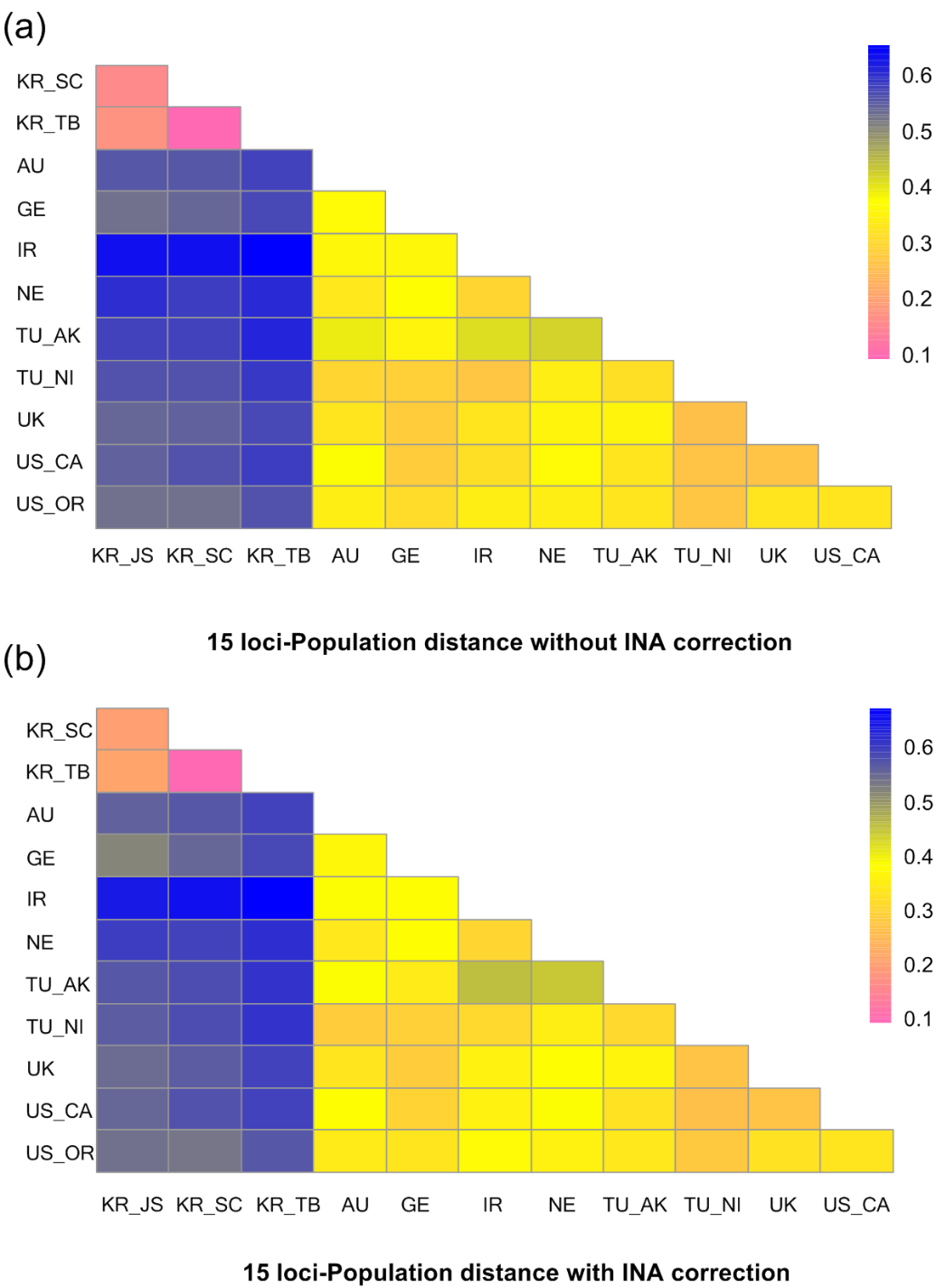


**Fig. S6** *D_C_* distance heatmaps among 12 *Heterodera schachtii* populations (a) without INA correction and (b) with INA correction. INA: including null alleles correction.

**(The following figures show the phylogenetic and clustering results based on 13 loci)**


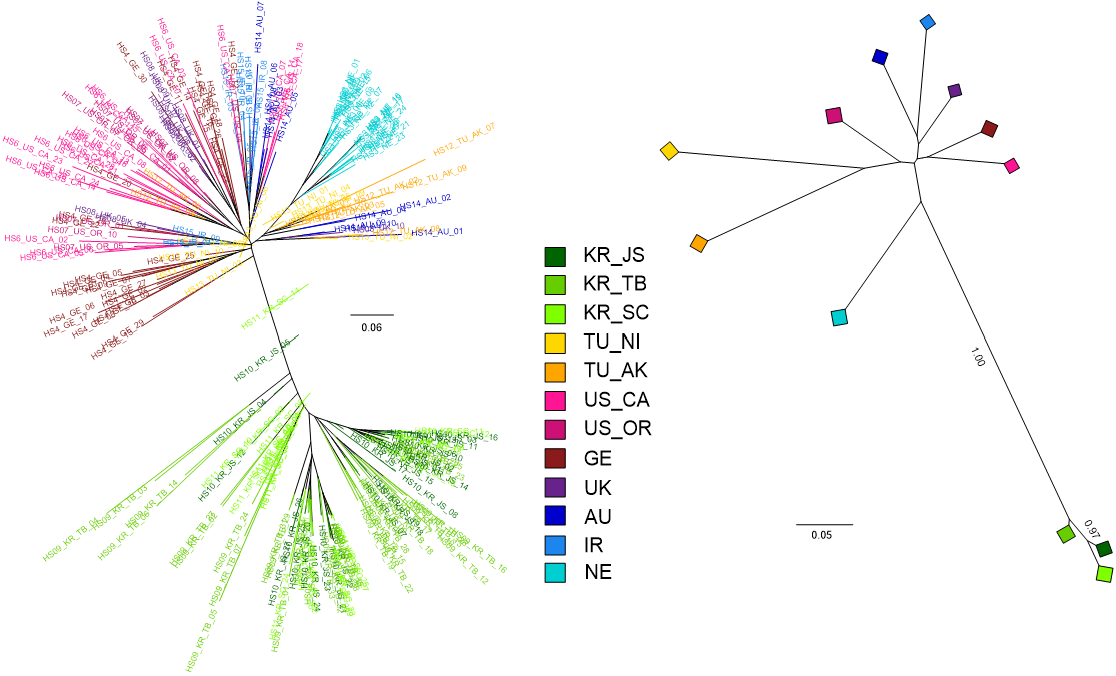


**Fig. S7** Phylogenetic trees displaying the relationship (a) among all *Heterodera schachtii* individuals based on Provesti’s distance and (b) among all populations based on *D_C_* distance of 13 microsatellite loci.


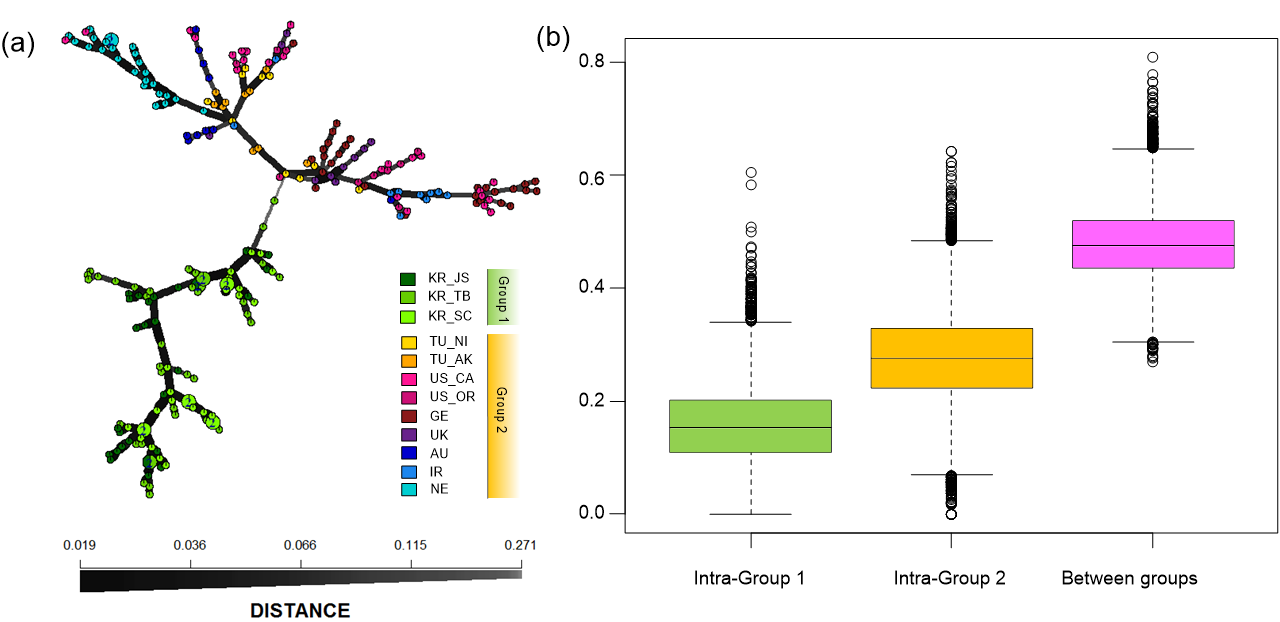


**Fig. S8** (a) Minimal spanning network displaying the topology among all the multilocus genotypes (MLGs) of 13 microsatellite loci based on Bruvo’s distance; (b) boxplots of Bruvo’s distances (y-axis) among MLGs of groups 1 (Intra-Group 1) and 2 (Intra-Group 2) and between MLGs of groups 1 and 2 (Between groups).


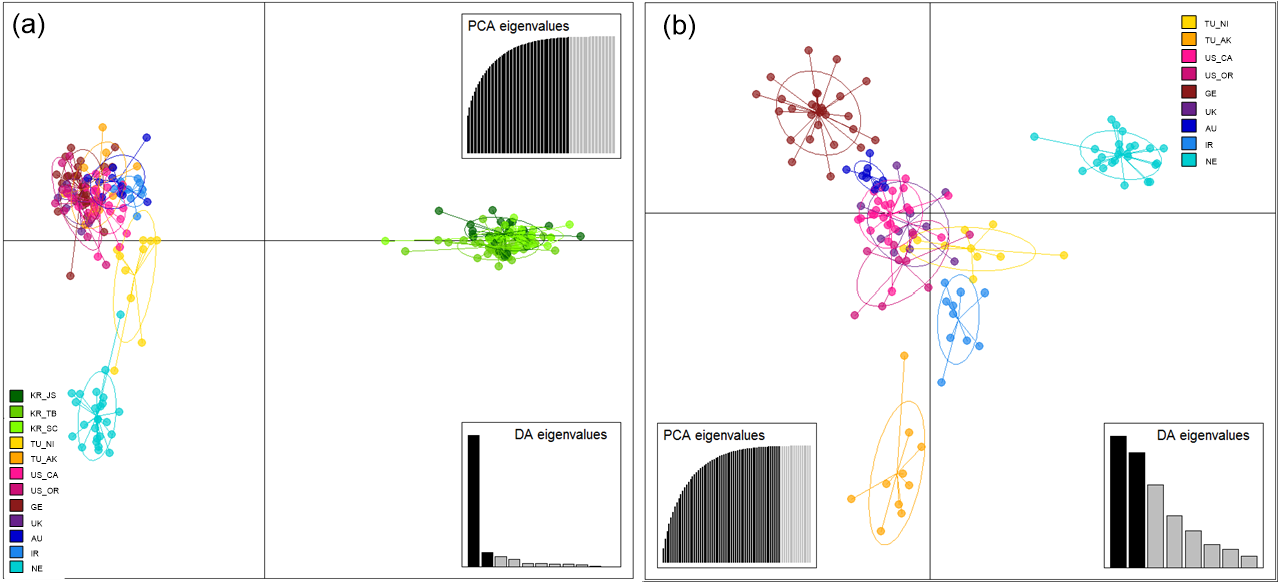


**Fig. S9** Population clusters based on discriminant analysis of principal components along with discriminant analysis (DA) eigenvalues and PCA eigenvalues retained for *Heterodera schachtii* individuals based on 13 microsatellite loci from (a) all 12 populations and (b) the nine populations when the Korean populations are excluded.

**Species identity of *Heterodera schachtii*:**

To explicitly confirm the identify of *Heterodera* species in this study (especially the divergent Korean populations, see results), we amplified the nuclear ITS1-5.8S-ITS2 region using primers TW81(F) (5’-GTTTCCGTAGGTGAACCTGC-3’) and AB28 (R) (5’-ATATGCTTAAGTTCAGCGGGT-3’) or 5.8SM5 (R) (5’-GGCGCAATGTGCATTCGA-3’) (Zheng et al. 2000) for 73 *Heterodera* individuals including *H.* *schachtii,* *H. betae*, *H. trifolii*, *H. glycines*, *H. avenae* and *H. filipjevi*. A total of 26 *H. schachtii* specimens from German, American and three Korean populations were sequenced. The final alignment is 951 bp long (including indels), and the neighboring-join tree (Fig. S10, below) reconstructed in MEGA 7 shows that the Korean individuals clustered with the German and American *H. schachtii*. These three countries formed a clade that was separated from other closely related *Heterodera* species, *H. betae* and *H. trifolii*. For the 26 ITS1-5.8S-ITS2 sequences of *H.* *schachtii*, five variable sites (including two indels) were discovered and defined six alleles. However, there were no diagnostic sites that could distinguish the Korean individuals from American and German samples. Our BLAST reports using the ITS1-5.8S-ITS2 sequences of the samples in this study also confirm the species identity of *H. schachtii*, with 99% and 100% matches to the *H. schachtii* ITS1-5.8S-ITS2 sequences in the National Center for Biotechnology Information (NCBI).


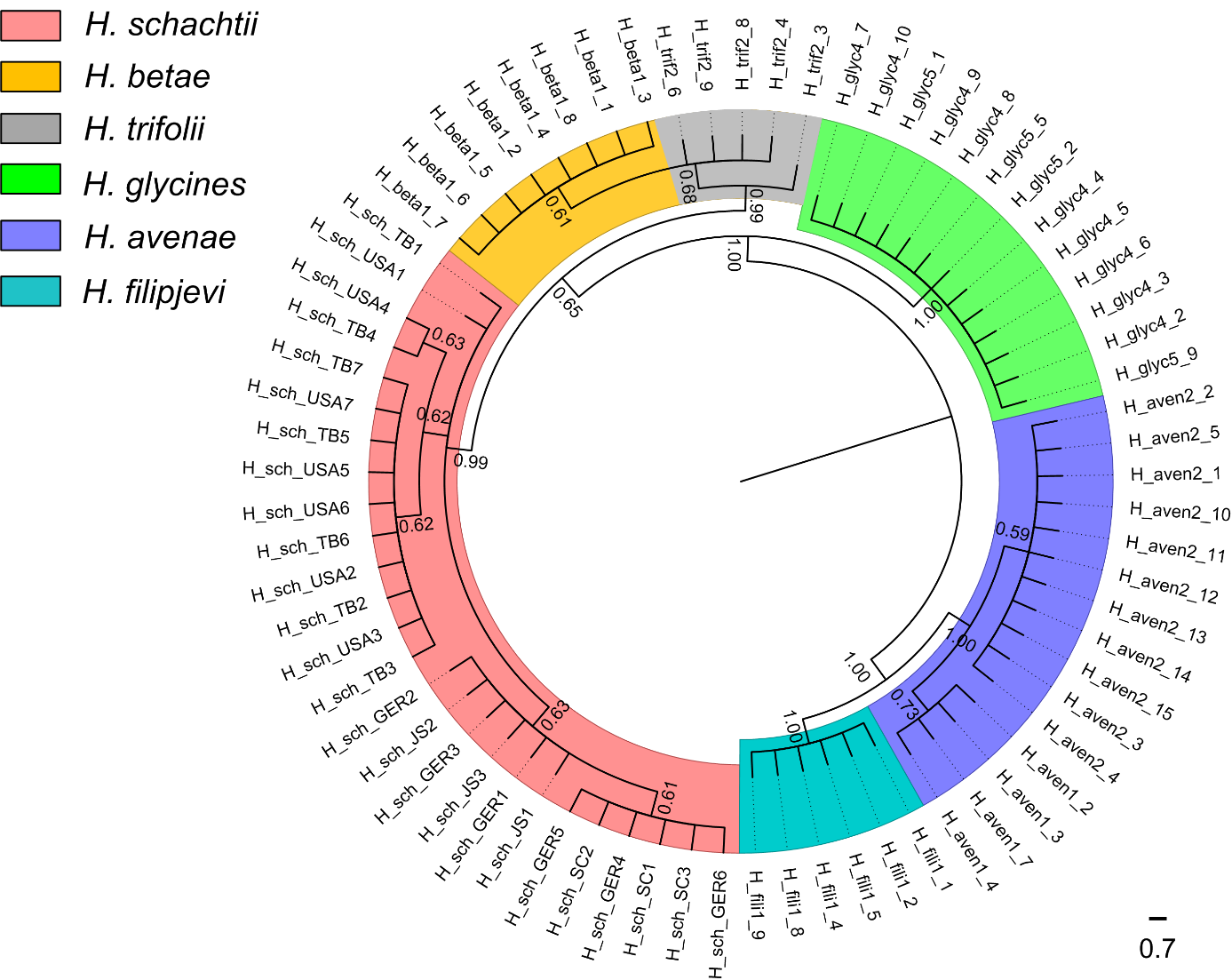


**Fig. S10** the unrooted neighboring-join tree showing the phylogeny among six *Heterodera* species based on ITS1-5.8S-ITS2 sequences of 73 individuals. Support for each node is indicated when the bootstrap value is > 50%. For *H. schachtii*, the five populations included were the American population (H_sch_USA), German population (H_sch_GER), and three Korean populations from Taeback (H_sch_TB), Samcheok (H_sch_SC), and Jeongseon (H_sch_JS).
